# Monogenic Diabetes: A Diagnostic Algorithm for Clinicians

**DOI:** 10.3390/genes4040522

**Published:** 2013-09-26

**Authors:** Richard W. Carroll, Rinki Murphy

**Affiliations:** 1 Endocrine, Diabetes and Research Centre, Wellington Regional Hospital, Private Bag 7902, Newtown, Wellington 6021, New Zealand; E-Mail: richard.carroll@ccdhb.org.nz; 2 Department of Medicine, University of Otago, Newtown, Wellington 6021, New Zealand; 3 Department of Medicine, Faculty of Medical and Health Sciences, University of Auckland, Private Bag 92019, Auckland, New Zealand

**Keywords:** monogenic, diabetes, maturity onset diabetes of the young, neonatal diabetes, genetic testing

## Abstract

Monogenic forms of beta cell diabetes account for approximately 1%–2% of all cases of diabetes, yet remain underdiagnosed. Overlapping clinical features with common forms of diabetes, make diagnosis challenging. A genetic diagnosis of monogenic diabetes in many cases alters therapy, affects prognosis, enables genetic counseling, and has implications for cascade screening of extended family members. We describe those types of monogenic beta cell diabetes which are recognisable by distinct clinical features and have implications for altered management; the cost effectiveness of making a genetic diagnosis in this setting; the use of complementary diagnostic tests to increase the yield among the vast majority of patients who will have commoner types of diabetes which are summarised in a clinical algorithm; and the vital role of cascade genetic testing to enhance case finding.

## 1. Introduction

**Figure 1:**
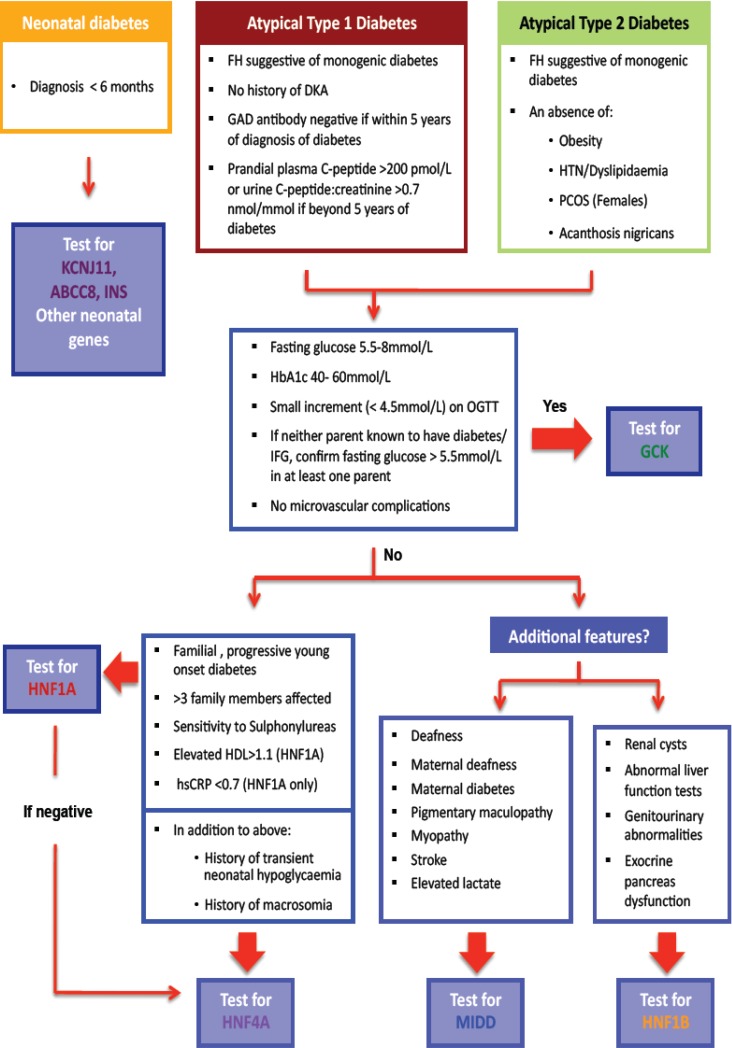
Diagnostic algorithm for assessment of suspected monogenic diabetes: diabetes diagnosed at <35 years of age.

Monogenic forms of beta cell diabetes account for approximately 1%–2% of all cases of diabetes, yet remain underdiagnosed [1,2]. Overlapping clinical features with common forms of diabetes makes diagnosis challenging [1,3,4,5]. We describe those types of monogenic beta cell diabetes which are recognisable by certain clinical features and have implications for altered management, the cost effectiveness of making a genetic diagnosis in this setting, the use of complementary diagnostic tests to increase the yield of positive monogenic diabetes diagnoses which are summarised in a clinical algorithm (Figure 1), and the vital role of cascade genetic testing to enhance case finding. 

## 2. Overview of Monogenic Beta Cell Diabetes Associated with Recognizable Phenotypes (Table 1)

The pancreatic beta cell is fundamental to the process of maintaining glucose homeostasis (critical for the normal functioning of most human tissues), by matching insulin secretion to ambient glucose. Glucose is “sensed” via a chain of glucose dependent, rate limiting intracellular steps that result in the metabolism of glucose to generate adenosine triphosphate (ATP). Rising ATP levels trigger closing of the ATP-sensitive potassium (K_ATP_) channel causing depolarization of the pancreatic beta cell enabling the secretion of synthesized insulin. Single gene defects in any step of these pathways produce monogenic beta cell diabetes [6].

### 2.1. Monogenic Diabetes that Present in Infancy

Neonatal diabetes mellitus (NDM) refers to diabetes diagnosed in the first 6 months of life, and has an estimated incidence of around 1 in 200,000 live births [3,7,8]. Historically, patients were treated with insulin as they were assumed to have type 1 diabetes, but the discovery in 2004 of the causal role of mutations in the K_ATP_ channels in many affected individuals, and the successful transfer of these patients from insulin to oral sulphonylurea therapy with improved control and less frequent hypoglycaemia, has led to the recommendation of genetic testing in all cases of neonatal diabetes [1,9,10,11,12].

NDM may be permanent (PNDM) or transient (TNDM), in which case the diabetes may remit spontaneously within 1–18 months (although relapse to permanent diabetes later in life is common) [1,11,13]. 50% of cases of PNDM and 20% of TNDM are estimated to result from activating mutations of the K_ATP_ channel genes (*KCNJ11 or ABCC8*) encoding the Kir6.2 and SUR1 subunits respectively [9,10,11]. Activating mutations in the K_ATP_ channels impair the ability of ATP (generated by pancreatic glucose metabolism), to close the channel, thereby preventing beta cell depolarization and insulin secretion. Consequently, these patients present with diabetic ketoacidosis or marked hyperglycaemia, and low levels of circulating endogenous insulin [11]. Orally administered sulphonylurea drugs, commonly used to treat type 2 diabetes, close the K_ATP_ channel by an ATP independent route and thus increase insulin secretion, although the required dose is frequently considerably higher. Unlike in type 2 diabetes however, patients with NDM due to K_ATP_ channel mutations who are treated with sulphonylurea therapy have near normalization of glycated hemoglobin (HbA1c) without significant hypoglycaemia, suggesting that insulin secretion is well regulated [1,12]. This phenomenon may be explained by the specific nature of the pathophysiological defect in NDM; unlike type 2 diabetes (where multiple factors promote glycaemic dysfunction), other physiological regulators of insulin secretion (glucagon like peptide 1, GLP-1, *etc.*) function normally.

In addition to NDM, 20% of patients with K_ATP_ channel mutations have associated neurological symptoms including Developmental delay, Epilepsy, Neonatal Diabetes “DEND” syndrome, which reflects the presence of extra-pancreatic K_ATP_ channels linking cell metabolism to electrical activity in muscle and brain [12,13]. Glibenclamide therapy (a non-beta cell selective Sulphonylurea) in these patients can ameliorate these extra-pancreatic manifestations also [14,15].

Many other genetic etiologies of NDM have been documented, some which are associated with extra-pancreatic manifestations [6,16,17,18]. Insulin therapy is essential for patients with other non-K_ATP_ channels forms of NDM.

**Table 1:** Characteristic Phenotypes of the commonly encountered diabetes subtypes, illustrating the clinically useful differences between type 1 and type 2 diabetes, and monogenic forms of diabetes.

Features associated with diabetes	Type 1 diabetes	Young onset Type 2 diabetes	Monogenic diabetes					
			GCK ^∗^	HNF1A ^#^	HNF4A ^#^	HNF1B ^#^	Neonatal diabetes	MIDD ^≠^
**DKA**	Yes	No	No	No ^∞^	No ^∞^	No	Yes	Yes/No
**Parent affected**	2%–4%	Yes	Yes ^≈^	Yes	Yes	Yes	variable	Mother
**Age of onset**	6 months to adulthood	Adolescence and young adulthood	Birth	Teens to young adulthood	Teens to young adulthood	Teens to young adulthood	<6 months	Young adulthood
**Obesity**	Population frequency	Increased frequency	Population frequency	Population frequency	Population frequency	Population frequency	Population frequency	Rare
**Glycaemic pattern**	Acute General hyperglycaemia	Progressive hyperglycemia	Stable, mild fasting glycaemia	Post-prandial hyperglycaemia initially, progressing to general hyperglycemia	Post-prandial hyperglycaemia initially, progressing to general hyperglycemia	Post-prandial hyperglycaemia initially Progressing to general hyperglycemia	Acute General hyperglycaemia	Variable dysglycaemic pattern either acute or slowly progressive
**β cell antibodies ^±^**	Yes	No	No	No	No	No	No	No
**C-peptide ^¥^**	Very low/Absent (>5 years)	Raised/Normal	Normal	Low but Detectable	Low but Detectable	Low but Detectable	Absent but detectable once treated with SU	Low but detectable
**hsCRP**	Normal	High/High normal	Normal	Very low	Normal	Normal	Normal	Normal
**Additional clinical features**	Other autoimmune disease (Thyroid, coeliac *etc*.)	Dyslipidaemia, PCOS, Hypertension, Acanthosis Nigricans	Absence of microvascular and macrovascular complications	Low renal threshold for glucose in early stages of diabetes	Macrosomia and transient neonatal hypoglycaemia	High renal involvement e.g., cysts *etc*.	Transient in 50% of cases, although may relapse	Deafness, short stature, macular dystrophy

* = Glucokinase; # = Hepatocyte nuclear factor; ≠ = Mitochondrial diabetes and deafness; ∞ = Excellent responses to Sulphonylurea therapy are commonly noted; ≈ = whilst the autosomal dominant inheritance pattern requires that at least one parent must be a carrier of the mutated gene, GCK mutations are frequently subclinical and an absence of a known family history of diabetes is not uncommon; ± = β cell antibodies are detected in approximately 90% of patients with type 1 diabetes at onset of dysglycaemia although the sensitivity declines later in the disease. Absent autoantibodies >5 years following onset are commonly seen in confirmed type 1 diabetes. Conversely, a small number of patients with type 2 diabetes and monogenic diabetes will have one or more detectable ß cell antibodies; ¥ = PCOS = Polycystic ovary syndrome.

### 2.2. Monogenic Diabetes Detected in Adolescence or Adulthood

#### 2.2.1. Glucokinase Monogenic Diabetes

The enzyme glucokinase (GCK), which phosphorylates glucose, functions as a sensor of ambient glucose levels [19]. The effect of a heterozygous inactivating mutation in *GCK* is mild fasting hyperglycaemia (5.5–8.0 mmol/L), minimal or normal post meal time glucose excursions (<3.0 mmol/L glucose during an oral glucose tolerance test), and an HbA1c level within the normal or slightly elevated range (usually <60 mmol/mol) [1,20,21,22]. GCK monogenic diabetes is thought to represent 20%–30% of all cases of monogenic diabetes and is inherited in an autosomal dominant pattern. Very rarely, severe NDM can result from homozygous mutations in *GCK*, arising from both parents having a heterozygous *GCK* mutation, more likely in cases of consanguinity [23,24].

Although the characteristic pattern of mild hyperglycaemia is present since birth, most cases are detected later in life during incidental glucose screening, often mistakenly diagnosed and treated as either type 1 or type 2 diabetes. Since microvascular complications are extremely rare in these patients, the confirmation of *GCK* mutation allows glucose lowering therapy to be stopped and carries a favourable prognosis without risk of progression of diabetes [1,21,22,25,26].

During pregnancy, women with a *GCK* mutation have a 50% chance of carrying a baby without a *GCK* mutation, in which case there is an increased risk of macrosomia and its obstetric consequences. Thus, maternal insulin treatment is indicated [27,28,29]. Conversely, if the mother carries a baby with a *GCK* mutation, no treatment is required [29]. Ultrasonographic monitoring of foetal size is currently recommended to decide whether or not to lower maternal glycaemia with insulin during pregnancy [30], although foetal genotyping using maternal blood sampling during early pregnancy may be available in the future.

#### 2.2.2. Hepatocyte Nuclear Factor (HNF) Monogenic Diabetes

The hepatocyte nuclear factor (HNF) family of proteins are transcription factors, required for the correct functioning of pancreatic beta cells [31]. 3 HNF subtype mutations are most commonly associated with monogenic diabetes: *HNF-1A*, *HNF-4A*, *HNF-1B*, with *HNF-1A* mutations being most common. Each heterozygous, loss of function mutation is inherited in an autosomal dominant fashion and results in early and progressive beta cell dysfunction, with diabetes presenting in late childhood or early adulthood [1,32,33]. The typical earliest manifestation is post-prandial hyperglycaemia with fasting normoglycaemia, eventually progressing to frank diabetes [1,33]. *HNF4A* is also associated with increased birthweight and a tendency to neonatal hypoglycaemia, which is thought to reflect fetal hyperinsulinemia and the differential roles for *HNF4A* in fetal and adult beta cells [34]. The risk of micro and macrovascular complications is comparable to type 1 and type 2 diabetes and correlates with glycaemic control [35].

Extrapancreatic roles of these transcription factors reflect their specific phenotypes which serve as useful diagnostic clues: *HNF1A* is also expressed in the renal tubule and *HNF1A* mutations result in impaired tubular glucose reabsorption, thus manifesting glycosuria at blood glucose levels <10 mmol/L (low renal glucose threshold) [1,5,30,33]. Early expression of *HNF1B* is seen in the kidney, liver, genital tract, lung, gut and pancreas [36]. Renal involvement is most consistently described in *HNF1B* mutation carriers due to abnormal renal development, and includes renal cysts, familial hypoplastic glomerulocystic kidney disease, atypical familiar hyperuricemic nephropathy, single and horseshoe kidney [36]. The penetrance of diabetes in patients with *HNF1B* mutations is unknown and may present in late adult life. Thus, annual diabetes screening is recommended. [36]. Other clinical features include genital tract malformations, abnormal liver function tests, pancreatic atrophy and exocrine insufficiency, gout and hyperuricemia.

Patients with *HNF-1A* and *HNF-4A* frequently demonstrate excellent and durable responses to low dose sulphonylurea therapy, although in some cases higher doses or additional therapy may be required (both Metformin and DPP4 inhibitors are occasionally added in those experiencing failure of sulphonylurea monotherapy, although there is no evidence to guide the use of these medications) [37,38]. Thus, insulin therapy can be avoided for many years after diagnosis in most cases, which has clear practical, social, occupational and health cost benefits. Insulin therapy is usually required in those with *HNF1B* diabetes due to pancreatic atrophy.

#### 2.2.3. Maternally Inherited Diabetes and Deafness

Maternally inherited diabetes and deafness (MIDD) accounts for up to 50% of cases of monogenic diabetes and up to 1% of unselected cases of diabetes [39]. The mt.3243A>G gene mutation is the most common cause of MIDD and affects tRNALeu(UUR) production within the mitochondria [39,40]. Because oocytes (but not spermatozoa) contribute mitochondria to the developing embryo, only females pass mitochondrial mutations to their children, (maternal inheritance). Mitochondria are responsible for cellular ATP production, and therefore mutations in its DNA should result in dysfunction within all tissues that are particularly metabolically active. However, it is clear that certain mitochondrial mutations are associated with tissue specific dysfunction (as seen with mt.3243A>G mutations resulting in MIDD) although the mechanisms remain to be fully elucidated. Progressive beta cell failure is seen in those who harbour mutations although the rapidity of insulin production failure is variable [39,41]. Carriers may also suffer from deafness and macular dystrophy (most commonly) but also strokes and myopathies. Insulin treatment for diabetes is frequently required. Metformin is theoretically contraindicated in MIDD due to concerns about lactic acidosis [39,42]. Coenzyme Q10 and thiamine replacement is advised to reduce progression of diabetes and hearing loss in the early stages of the disorder [39,43].

### 2.3. Monogenic Diabetes due to Rare Etiologies

Collectively, less than 5% of cases of early-adult onset monogenic diabetes may be caused by mutations in genes most commonly associated with permanent neonatal diabetes such as *ABCC8*, *KCNJ11*, *IPF1*, *NEUROD1*, *CEL* and *INS* [17,44]. Most of these cases have been found by screening parents of affected cases with PNDM. Approximately 15% of cases of maternally inherited diabetes due to a mitochondrial genetic cause are estimated to be caused by mitochondrial point mutations at positions other than 3243 [39]. Direct sequencing of the mitochondrial genome are required to detect many of these rare mutations.

## 3. Diagnosing Monogenic Diabetes Is Cost Effective

Genetic testing becomes more cost effective as the cost of the test decreases, the likelihood of detecting a monogenic cause increases, and if monogenic diagnosis results in cost savings or other benefits in patient health care. It should be noted however that evidence for the cost effectiveness of making a diagnosis of monogenic diabetes is predominantly based on models with inherent assumptions; further study is required to quantify these results.

### 3.1. Neonatal Diabetes

More than 90% of children diagnosed with *KCNJ11* or *ABCC8* mutations are able to transfer from insulin to lifelong sulphonylurea therapy, and such conversions are associated with improvement in HbA1c sustained over many years reducing concomitant microvascular complications [12]. It has been estimated that, with an initial cost of genetic testing of US$2815 and 4 day cost of inpatient transfer of insulin to sulphonylurea therapy (assuming home blood glucose monitoring declines from 6 tests per day to 3 tests per day), the decline in expected probability of microvascular complications from an assumed lifetime mean HBA1c of 8.1% to 6.4% becomes cost saving by 10 years and increasing thereafter [45].

### 3.2. GCK, HNF1A and HNF4A

With a single cost of genetic testing of US$2000 for all 3 genes: *GCK*, *HNF1A*, *HNF4A* without phenotype discrimination, genetic testing was estimated to be cost saving once the diagnostic yield exceeded 20% [46]. This was based on a simulation model of type 2 diabetes complications occurring in incident cases of diabetes diagnosed in a hypothetical population aged 20–40 years according to UKPDS data, with an underlying prevalence of monogenic diabetes of 2% (35% with *GCK*, 65% with *HNF1A* or *HNF4A*), assuming 75% of those with *HNF1A* or *HNF4A* would be treated effectively with sulphonylurea monotherapy and that all patients with *GCK* would discontinue therapy.

## 4. Design of an Algorithm for the Diagnosis of Suspected Monogenic Diabetes

Given the present high cost of genetic testing a judicious approach to selecting those patients who may benefit from testing for monogenic diabetes is required, to increase the positive yield and improve cost-effectiveness of genetic testing. Although future perspectives of genetic diagnosis are moving towards enabling multiple genes to be tested in a single experiment though targeted-gene sequencing approaches, being clinically alert to when such testing might be required is likely to remain necessary.

### 4.1. The Importance of Atypical Features

The clinical algorithm (Figure 1) begins by emphasizing the consideration of atypical features that count against type 1 or type 2 diabetes, either when making a new diagnosis of diabetes or revisiting old diagnoses. In lean patients presenting at a young age when type 1 diabetes remains the more likely diagnosis, a family history of diabetes suggestive of autosomal dominant inheritance, or an absence of detectable antibodies directed against the beta cell should prompt consideration of a monogenic aetiology. An absence of a history of ketoacidosis (almost always the consequence of absolute insulin deficiency as usually demonstrable early after the diagnosis of type 1 diabetes in young patients) or when endogenous insulin production is still clearly evident (presence of post meal-time c-peptide levels measured in serum or urine), should further raise this suspicion [47]. 

The increased prevalence of obesity globally has significantly reduced the sensitivity of a defined upper limit of BMI when considering a monogenic diagnosis. However, the clinician should be wary of confirming a diagnosis of type 2 diabetes in the absence of other stigmata of insulin resistance such as polycystic ovary syndrome, dyslipidemia, or hypertension, particularly in the context of low endogenous insulin secretion (very high insulin levels expected commensurate with significant insulin resistance in type 2 diabetes) [48]. Conversely, the presence of acanthosis nigricans, although rare, counts against a monogenic cause for diabetes.

The MODY probability calculator estimates the pre-test probability of *HNF1A*, *HNF4A*, and *GCK* monogenic diabetes collectively using the following variables: age at diabetes diagnosis, gender, current glucose lowering therapy, time to insulin, BMI, HbA1c, age and parental diabetes status [49]. This calculator was validated using a predominantly caucasian, UK based population; the applicability to a New Zealand population is not known, and will be the subject of future study. Our algorithm (Figure 1) prompts the clinician to consider a wider range of clinical and biochemical features to direct testing to the most likely genetic subtype and improve diagnostic yield.

### 4.2. Selecting the Most Likely Gene(s) for Testing

Once a diagnosis of monogenic diabetes is considered possible, the clinician is left with number of genetic tests that could be requested, each of which carries a cost. Furthermore, the prevalence of each monogenic form of diabetes differs such that the diagnostic yield is increased by a testing process that initially focuses on the most common subtype. The algorithm was thus designed to direct the clinician along a process of exclusion, so that rarer forms of monogenic diabetes would only be considered once commoner forms were excluded. *GCK* monogenic diabetes accounts for up to 50% of cases of monogenic diabetes and often presents with a very typical biochemical phenotype [1,2,3,4,5]. Thus, the guidelines direct the clinician to consider this option first. Thereafter, clinicians are invited to consider *HNF1A* before *HNF4A* (due to frequency), and finally to consider *HNF-1B* mutations or mitochondrial disorders if there are specific extra-pancreatic features such as renal disease or deafness respectively in addition.

### 4.3. The Use of Additional Biochemical Tests May Aid in Diagnosis (See Table 1)

#### 4.3.1. C-Peptide

C-peptide, a cleaved remnant of proinsulin, is secreted by the beta cell at concentrations equivalent to insulin release, and therefore serves as a surrogate marker of endogenous insulin production. The measurement of C-peptide is particularly helpful when distinguishing between type 1 diabetes and monogenic diabetes, as an undetectable serum C-peptide is usually seen after 5 years of T1D disease duration (although approximately 8% of those with type 1 diabetes will exhibit low volume residual endogenous insulin production beyond 5 years) [50]. Persistent endogenous insulin production (as indicated by post prandial C-peptide levels >200 pmol/L (when glucose is >8 mmol/L) or postprandial urinary C-peptide creatinine ratio ≥0.7 nmol/mmol) beyond 5 years of diabetes diagnosis makes type 1 diabetes unlikely [50,51]. It should be remembered that there is marked variability in the rapidity of beta cell failure in type 1 diabetes and therefore C-peptide levels may remain within the normal range early after the diagnosis (*i.e.*, the honeymoon period where insulin therapy requirements are low). In a suspected monogenic diabetes case who has recently been diagnosed with diabetes, testing the parent with diabetes for persistent C-peptide could be helpful in supporting a monogenic aetiology.

#### 4.3.2. Islet Cell Autoantibodies

98% of patients with a new diagnosis of type 1 diabetes will have at least one detectable islet cell autoantibody (anti-GAD, IA-2, IAA, ZnT8A), and 70% will have detectable levels of at least one islet cell autoantibody at 11 years post diagnosis [52]. The prevalence of these antibodies in those with *HNF1A*, *GCK* or *HNF4A* is low at 1% (comparable to control subjects) [53]. Thus the presence of detectable islet cell autoantibodies counts strongly against a diagnosis of monogenic diabetes, and genetic testing should only be performed in this context if there is overwhelming evidence otherwise to support the diagnosis.

#### 4.3.3. Highly Sensitive CRP

Measuring hsCRP may be particularly helpful in discriminating between *HNF1A* and other forms of diabetes. HNF-1A binding sites are located at promoter sites in the gene coding for C-reactive peptide (CRP). Consequently, markedly lower levels of high sensitivity CRP (hsCRP) are seen in monogenic diabetes as a result of an *HNF1A* mutation than in other forms of diabetes [54]. The highest discriminatory value of hsCRP is between *HNF1A* and T2D, although hsCRP values are also higher among those with *HNF4A*, *GCK*, or *HNF1B* than HNF1A. However, the modest test performance (sensitivity 79%, specificity 70%) requires that additional clinical characteristics are considered in combination [55]. The hsCRP is not useful if elevated (>10 mg/L) as this usually indicates the presence of confounding inflammation, and should be repeated after a few weeks.

#### 4.3.4. High-Density Lipoprotein (HDL)

Insulin resistance is characteristically associated with a reduction in circulating HDL levels. Thus, normal or elevated HDL levels indicate that insulin resistance may not be a major component of the disease (*i.e.*, type 2 diabetes is less likely). The HDL level displays moderate discrimination when distinguishing between *HNF1A* carriers and those with type 2 diabetes, with a plasma HDL >1.12 mmol/L favouring a diagnosis of monogenic diabetes (75% sensitive and 64% specific) [56]. HDL levels are similar between *HNF1A*, T1D and healthy controls.

## 5. Cascade Genetic Testing of Family Members

The key diagnostic challenge in monogenic diabetes is the detection of the index case (first individual diagnosed with monogenic diabetes in the family). Once this person has been identified, this is the starting point for family tracing or cascade genetic testing by which the majority of familial cases of monogenic diabetes can be efficiently detected and confirmed in the most cost-effective manner. Given the increasing prevalence of type 2 diabetes, the presence of diabetes in family members with monogenic diabetes cannot be assumed to be of the same etiology, although the probability is much higher. Co-ordinated cascade genetic testing should be organised through referral of the index case to the genetics service in collaboration with the diabetes service. Risk notification, informing first and then second degree relatives that (**1**) they are at risk of monogenic diabetes (**2**) this type of diabetes may have implications for their diabetes therapy and general health (**3**) genetic testing is available to clarify if they do or do not have monogenic type of diabetes is important. All cases detected this way will become index cases for risk notifcation of their own first and second degree relatives, maximising cost-effectiveness of genetic testing and monogenic diabetes case finding.

## 6. Conclusions

Monogenic diabetes should be considered in the differential diagnosis of diabetes. Vigilance for atypical features among those classified as having either type 1 or type 2 diabetes along with a systematic clinical approach, should increase the yield from targeted diabetes genetic testing and ultimately improve clinical outcomes.
